# The Evolution of Academic Performance in Nine Subspecialties of Internal Medicine: An Analysis of Journal Citation Reports from 1998 to 2010

**DOI:** 10.1371/journal.pone.0048290

**Published:** 2012-10-31

**Authors:** Yan Zhang, Jia Kou, Xue-Guang Zhang, Li Zhang, Shu-Wen Liu, Xue-Ying Cao, Yuan-Da Wang, Ri-Bao Wei, Guang-Yan Cai, Xiang-Mei Chen

**Affiliations:** Department of Nephrology, State Key Laboratory of Kidney Diseases, Chinese PLA General Hospital, Beijing, People’s Republic of China; Technical University of Denmark, Denmark

## Abstract

**Background:**

Internal medicine includes several subspecialties. This study aimed to describe change trend of impact factors in different subspecialties of internal medicine during the past 12 years, as well as the developmental differences among each subspecialty, and the possible influencing factors behind these changes and differences.

**Methods:**

Nine subspecialties of internal medicine were chosen for comparison. All data were collected from the Science Citation Index Expanded and Journal Citation Reports database.

**Results:**

(1) Journal numbers in nine subspecialties increased significantly from 1998 to 2010, with an average increment of 80.23%, in which cardiac and cardiovascular system diseases increased 131.2% rank the first; hematology increased 45% rank the least. (2) Impact Factor in subspecialties of infectious disease, cardiac and cardiovascular system diseases, gastroenterology and hepatology, hematology, endocrinology and metabolism increased significantly (p<0.05), in which gastroenterology and hepatology had the largest increase of 65.4%. (3) Journal impact factor of 0–2 had the largest proportion in all subspecialties. Among the journals with high impact factor (IF>6), hematology had the maximum proportion of 10%, nephrology and respiratory system disease had the minimum of 4%. Among the journal with low impact factor (IF<2), journal in nephrology and allergy had the most (60%), while endocrinology and metabolism had the least (40%). There were differences in median number of IF among the different subspecialties (p<0.05), in which endocrinology and metabolism had the highest, nephrology had the lowest. (4) The highest IF had a correlation with journal numbers and total paper numbers in each field.

**Conclusion:**

The IF of internal medicine journals showed an increasingly positive trend, in which gastroenterology and hepatology increase the most. Hematology had more high IF journals. Endocrinology and metabolism had higher average IF. Nephrology remained the lowest position. Numbers of journals and total papers were associated with the highest IF.

## Introduction

Many subspecialities have developed from general internal medicine since the 1970s. Internal medicine kept the unifying requirement of training in general internal medicine but underwent just as much subspecialization during that time. Independent status for some of the subspecialties seems inevitable. The subspecialties prosper, although unevenly, and retain varying degrees of connection to their internal medicine roots. Many subspecialties of internal medicine are distinguishable by the specialized knowledge and understanding of sophisticated technologic procedures required for each. Presently, there are no gold standards for evaluating different subspecialties, because particular specialties are compared independently of their sizes. It is difficult to estimate and compare the differences of development trend in various subspecialties among different subspecialties.

With the development of bibliometrics, and the extensive description of Science Citation Index (SCI), impact factor has been a widely used measurable indicator [1]. A journal’s impact factor is calculated by dividing the total number of published citations to articles in the journal during the previous 2 years by the number of source items (original research articles, review articles, etc) published by the journal during the same 2 years. Every year, the Institute for Scientific Information publishes impact factors for 5,000 science and technology journals. Although there are controversial aspects of using impact factors to assess academic output, because of the imbalance of development among various fields, at present, it is being used to judge the quality of a journal, a researcher or medical scientist as well as the quality of their institution. It has become an imperative for scientists to publish their work in journals with a high impact factor in order to become widely recognized [2].

In this study, we described the change trend in each subspecialty of internal medicine, then we tried to investigate the following questions: 1, Are there difference of journal impact factors among subspecialties? 2, what are the possible influencing factors behind these differences? In order to answer these questions, our study analyzed the change of impact factor of nine subspecialties journals in internal medicine field during the recent 12 years, including allergy, infectious disease, respiratory system disease, cardiac and cardiovascular system disease, gastroenterology and hepatology, hematology, rheumatology, nephrology, endocrinology and metabolism. The compositions of journal impact factor in each subspecialty were compared. The associated influencing factors were analyzed. To our knowledge, this is the first study to examine the difference among subspecialties in internal medicine from a perspective concerning impact factor and their respective characteristics.

## Materials and Methods

### 1.1 Journals Selection and Inclusion Criteria

Journal impact factor of nine subspecialties in internal medicine field were analyzed from 1998 to 2010, including allergy, infectious disease, respiratory system disease, cardiac and cardiovascular system disease, gastroenterology and hepatology, hematology, rheumatology, nephrology, endocrinology and metabolism.

Internal medicine journals enrolled in this study fulfills the following criteria: (i) the journal of each subspecialty was listed in the category of Science Citation Index Expanded (SCIE) subject categories by the Institute for Scientific Information (ISI); (ii) the journal was listed in the Journal Citation Reports (JCR) and has an impact factor (IF) from 1998 to 2010. (iii) Pure surgery, pediatrics, geriatrics or nursing journals were excluded by a consensus of the authors, such as *Digestive Surgery, Journal of Gastrointestinal Surgery, Pediatric Pulmonology, Journal of Pediatric Hematology Oncology, American Journal of Geriatric Cardiology, Gastroenterology Nursing, Nephrology Nursing Journal*, et al. There were totally 78 journals excluded based on the criteria.

Data on journals in each category were collected, including journal title, impact factor for each year, total number of journals for each year. Although some journals have been renamed, journals with same International Standard Serial Number (ISSN) were regarded as one journal.

### 1.2 Impact Factor Analysis

To investigate the change trend in each subspecialty of internal medicine and to compare the quality of nine subspecialties journal, the retrieved journals were analyzed based on the impact factors generated according to JCR from 1998 to 2010 established by the ISI. Besides cooperation of the changes of all subspecialty journals, three factors were evaluated: higher impact factors (IF>6), lower impact factors (IF<2), and median number of impact factor of each subspecialty journals.

For each journal, impact factors available for the years 1998 to 2010 were determined. Because of the method of calculating the impact factor, the 2010 impact factors were the latest available during July 2011.

The methods we used to ascertain the database and classify the records involved highly trained personnel, independent evaluation, and third-party arbitration of differences between reviewers.

#### 1.2.1 Grade analysis of journal impact factors of subspecialties in internal medicine field

In order to determine if there are differences of journal impact factors among nine subspecialties, Journal impact factors of different subspecialties were divided into four grades with semi-quantitative level, including <2, 2–4, 4–6, and >6, to establish the grade variable data. Distributions of journal impact factors of different subspecialties in each grade were compared.

#### 1.2.2 Analysis of the minimum, median, maximum and percentiles of journal impact factor

Analysis of the minimum, median, maximum and percentiles of journal impact factor were introduced as another method to determine the differences of journal impact factors among subspecialties. We used information from the JCR database of the ISI to identify the impact factor of subspecialty journals at various points of the distribution of the impact factor (maximum, median, minimum, and 97.5th, 75th, 25th, and 2.5th percentiles). The interquartile ranges were also calculated.

The median value of the journal impact factors in each subspecialty was used to make a further analysis. The average median impact factor was calculated to compare the difference among the subspecialties.

#### 1.2.3 Correlation analysis

We summarized the number of journals and papers published in nine subspecialties in 2010. Correlation analyses were made between the highest impact factor and the journal number and between the highest impact factor and the total number of papers publication in each field, which may provide clues regarding the possible influencing factors behind the differences among journals of subspecialties in internal medicine.

### 1.3 Statistical Methods

Statistical analyses were performed using SPSS 17.0 (SPSS Inc., Chicago, IL, USA). The Kruskal-Wallis test along with the Wilcoxon rank-sum test was performed, comparing the median impact factors among nine subspecialty groups. The changes in median impact factors among these groups from 1998 to 2010 were also determined. The Correlation Analysis was performed to determine the significant relationship between the highest impact factor and total number of subspecialty journals or total number of published papers.

## Results

### (1) Changes of Journal Number in Each Subspecialty during the Recent 12-year Study Period

From 1998 to 2010, there has been a linear growth in the number of journal titles in nine subspecialties. An average increase is 80.23%, with the highest increase of 131.2% in the field of cardiac and cardiovascular system disease, the lowest increase of 45% in the field of hematology. Changes of journal number were as follows: allergy increased from 13 to 20, an increase of 53.8%; infectious disease from 29 to 53, an increase of 82.8%; respiratory system disease from 19 to 37, an increase of 94.7%; cardiac and cardiovascular system disease from 64 to 148, an increase of 131.2%; gastroenterology and hepatology from 32 to 60, an increase of 87.5%; hematology from 40 to 58, an increase of 45%; rheumatology from 14 to 26, an increase of 85.7%; nephrology from 20 to 38, an increase of 90%; endocrinology and metabolism from 74 to 112, an increase of 51.4%.

### (2) Changes Trend of Journal Impact Factors in Each Subspecialty of Internal Medicine during the 12 Years

From 1998 to 2010, all journal impact factors in each year showed a non-normal distribution. Rank-sum test was used to test the dynamic changes in nine subspecialties. Five subspecialties showed significant differences, such as infectious diseases (P = 0.01), cardiac and cardiovascular system disease (P = 0.000), gastroenterology and hepatology (P = 0.002), hematology (P = 0.002), endocrinology and metabolism (P = 0.000). However, no significant changes were found in allergy (P = 0.393), respiratory system disease (p = 0.496), rheumatology (p = 0.307) and nephrology (p = 0.835).

**Figure 1:**
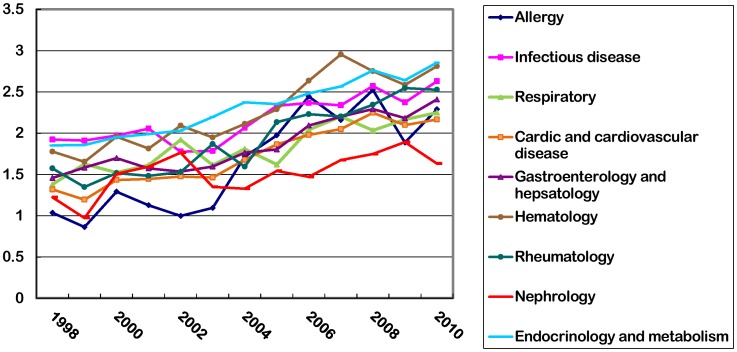
Dynamic change of median impact factors between 1998 and 2010.

Journals had significant increases in their median impact factors from 1998 to 2010. Allergy had a greatest increase by 121.14% in their median impact factor from 1998 to 2010, followed by gastroenterology and hepatology 65.29%, cardiac and cardiovascular system disease 64.29%, respiratory system disease 63.16%, rheumatology 60.55%, hematology 58.16%, endocrinology and metabolism 54.21%, infectious diseases 36.81% and nephrology 33.25%. (Figure 1).

### (3) Differences of Journal Impact Factors Among Subspecialties of Internal Medicine by Grade Analysis

**Figure 2:**
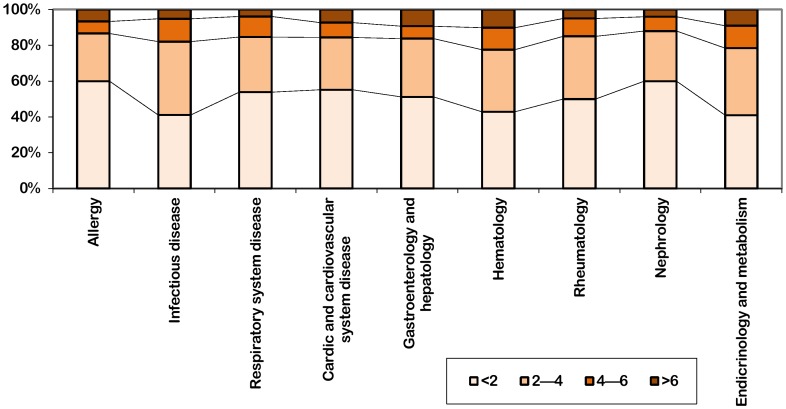
Comparison of constituent ratio of journal impact factors in nine subspecialties.

In order to determine if there are differences of journal impact factors among subspecialties, grade analysis of journal impact factor in various subspecialties was carried out as follows:

Journal impact factors of different subspecialties were divided into four grades by semi-quantitative level, including <2, 2–4, 4–6 and >6, to establish the grade variable data. Distributions of journal impact factors of different subspecialties in each grade were compared. By calculating the average constituent ratio from 1998 to 2010, most of journal impact factor in various subspecialties were less than 4, in which journal impact factor of 0–2 had the largest proportion in all subspecialties. (Figure 2).

We calculated the percentages related to journals in each subspecialty. Among the journals with high impact factor (IF>6), hematology had the maximum proportion account for 10% (5 journals), nephrology and respiratory system disease had the minimum with 4% (1 journal each). Other subspecialties were listed in descending order as follows: gastroenterology and hepatology 9.3% (4 journals), endocrinology and metabolism 9% (8 journals), cardiac and cardiovascular system disease 7.4% (7 journals), allergy 6.7% (1 journal), infectious disease 5.4% (2 journals), rheumatology 5.1% (1 journal). The order of journal numbers in each subspecialties with high impact factor (IF>6) remained unchanged till 2010, in which hematology had the maximum and nephrology had the minimum.

Among the journal with low impact factor (IF<2), journal of nephrology and allergy had the most (60%, 15 journals in nephrology and 9 journals in allergy), while endocrinology and metabolism had the least (40%, 36 journals). Other subspecialties were listed in descending order as follows: cardiac and cardiovascular system disease 55.8% (53 journals), respiratory system disease 56% (14 journals), gastroenterology and hepatology 51.2% (22 journals), rheumatology 50% (10 journals), infectious disease 42.1% (16 journals), hematology 42% (21 journals). In 2010, for the journal impact factors <2, nephrology still had the maximum proportion (60.5%), and infectious disease had the minimum (24.5%).

We analyzed dynamic changes of the proportion of low-impact factor journals (<2) to reflect the developmental trend of each subspecialty. By comparing the data within recent 12 years, constituent ratio of low impact factor journals in the field of infectious diseases, cardiac and cardiovascular system disease, gastroenterology and hepatology, hematology, endocrinology and metabolism were decreased gradually with significant differences (p<0.05). On the contrary, no significant differences were found in the journals of allergy, respiratory system disease, rheumatology and nephrology (Table 1).

**Table 1:** Comparison of constituent ratio of journal impact factors less than 2 from 1998 to 2010.

	AL		ID		RP		CV		GE		HM		RM		NP		EC	
IF	<2	≥2	<2	≥2	<2	≥2	<2	≥2	<2	≥2	<2	≥2	<2	≥2	<2	≥2	<2	≥2
1998	11	2	16	13	12	7	42	22	22	10	24	16	8	6	14	6	42	32
1999	11	2	15	14	12	7	47	25	24	12	24	19	10	15	13	7	43	35
2000	11	3	16	16	13	7	47	29	26	12	23	21	10	8	14	8	42	41
2001	9	5	16	17	12	8	56	29	24	16	25	21	12	7	13	9	42	41
2002	11	3	22	13	12	10	55	31	25	16	24	26	11	9	12	10	42	44
2003	9	4	22	14	15	9	54	34	24	17	26	24	11	9	15	9	37	39
2004	8	5	16	21	16	10	53	36	25	16	23	29	13	9	17	9	39	48
2005	7	6	15	24	15	11	47	44	23	19	20	29	11	11	16	11	35	53
2006	6	7	13	28	12	14	46	51	17	25	20	34	10	12	16	12	32	57
2007	5	8	16	25	13	14	46	49	17	27	18	33	8	12	19	10	27	61
2008	5	8	11	30	14	15	63	62	14	33	12	40	9	12	18	11	23	70
2009	11	10	19	36	15	20	61	64	24	32	19	38	8	14	20	15	31	73
2010	9	11	13	40	16	21	66	82	22	38	15	43	11	15	23	15	32	80
P	0.07		0.03		0.717		0.003		0.001		0.001		0.827		0.388		0.000	

AL  =  Allergy; ID  =  Infectious disease; RP  =  Respiratory system disease; CV  =  Cardiac and cardiovascular system disease; GE  =  Gastroenterology and hepatology; HM  =  Hematology; RM  =  Rheumatology; NP  =  nephrology; EC =  Endocrinology and metabolism.

### (4) Analysis of the Average Median Impact Factors in Each Subspecialty within 12 Years

**Figure 3:**
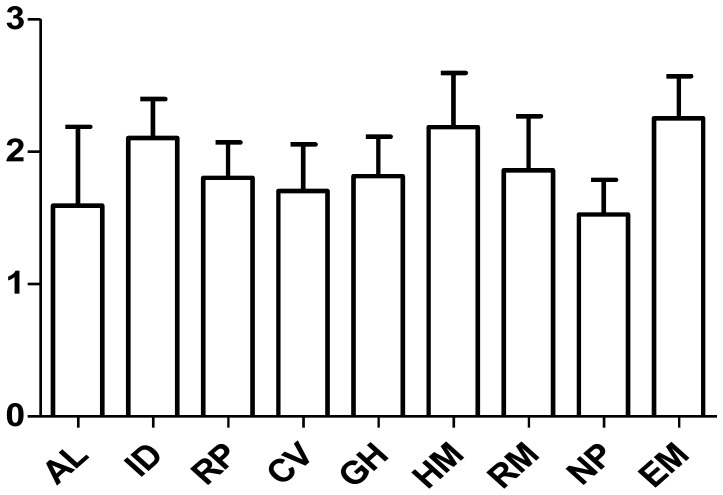
Analysis of the average median impact factors in each subspecialty from 1998 to 2010. AL = Allergy; ID = Infectious disease; RP = Respiratory system disease; CV = Cardiac and cardiovascular system disease; GE = Gastroenterology and hepatology; HM = Hematology; RM = Rheumatology; NP = nephrology; EC = Endocrinology and metabolism.

The median impact factors in each subspecialty showed a normal distribution. We found endocrinology and metabolism had the highest impact factor and nephrology had the lowest. The average median impact factors in each subspecialty were expressed in descending order as follows: endocrinology and metabolism (2.301±0.348), hematology (2.261±0.439), infectious disease (2.162±0.289), rheumatology (1.916±0.431), gastroenterology and hepatology(1.861±0.329), respiratory system disease (1.831±0.289), cardiac and cardiovascular system disease (1.725±0.358), allergy (1.647±0.603) and nephrology (1.515±0.250).There were significant differences among each groups (p = 0.001). (Figure 3).

### (5) Analysis of the Minimum, Median, Maximum and Percentiles of Journal Impact Factor in 2010

Data of impact factors in 2010 were compared in the journals of nine subspecialties. Among the highest impact factor journals, endocrine and metabolism had the highest value of 22.469; nephrology had the lowest value of 8.288. Among the lowest impact factor journals, cardiac and cardiovascular system disease had the lowest value of 0.000, gastroenterology and hepatology had the highest value of 0.311. Among the median impact factor in 2010, hematology was 2.812 with the highest value; nephrology was 1.631 with the lowest value. Interquartile range were biggest in rheumatism (3.141) and smallest in respiratory system disease (1.584), indicating a significant discrete trend in rheumatology (Table 2).

**Table 2:** Comparisons of journal impact factors at main points of the distribution in 2010.

Variable	NO.	Min	P2.5	P25	Median	P75	P97.5	Max	Interquartile range
AL	20	0.143	0.143	0.766	2.291	3.434	9.273	9.273	2.668
ID	53	0.265	0.294	1.986	2.631	3.781	13.359	16.144	1.795
RP	37	0.037	0.037	1.424	2.250	3.008	10.191	10.191	1.584
CV	148	0.000	0.120	1.235	2.167	3.505	9.653	14.429	2.27
GE	59	0.311	0.123	1.550	2.410	3.840	11.459	12.032	2.290
HM	58	0.033	0.388	1.575	2.812	4.642	12.590	14.429	3.067
RM	26	0.043	0.043	1.077	2.527	4.218	9.082	9.082	3.141
NP	38	0.023	0.023	1.154	1.631	2.975	8.288	8.288	1.821
EC	112	0.052	0.211	1.844	2.586	4.407	13.70	22.469	2.568

AL  =  Allergy; ID  =  Infectious disease; RP  =  Respiratory system disease; CV  =  Cardiac and cardiovascular system disease; GE  =  Gastroenterology and hepatology; HM  =  Hematology; RM  =  Rheumatology; NP  =  nephrology; EC =  Endocrinology and metabolism.

### (6) Correlation Analysis of Highest Impact Factor and Journal Number and Total Number of Papers in Each Field

**Figure 4:**
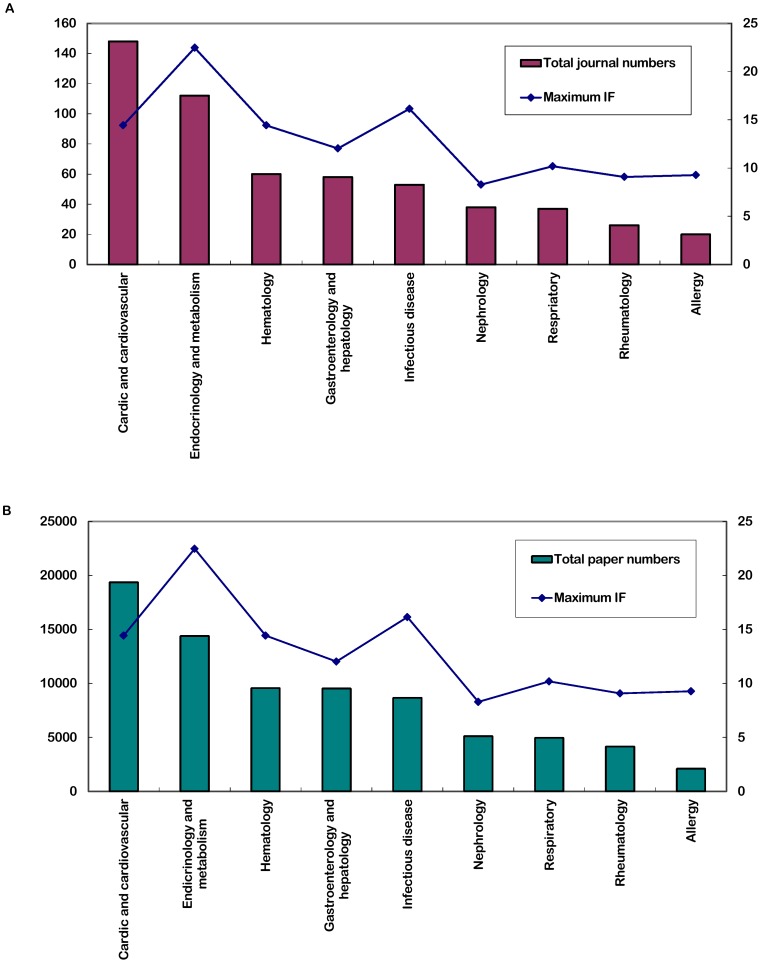
Correlation analysis of highest impact factor and journal number (a) and total number of papers in each field (b).

The highest impact factor, journal number and the total number of published papers showed a normal distribution. The highest impact factor in 2010 had a linear correlation with the journal number in each field (r = 0.684, p = 0.048). The highest impact factor in 2010 also had a linear correlation with the total number of papers publication (r = 0.704, p = 0.034) (Figure 4). That means journals that had much more numbers or more articles had greater increases in impact factors than journals with less numbers only or less articles only, respectively. This finding might become one of the probable influencing factors behind the differences among journals of subspecialties in internal medicine.

## Discussion

Internal medicine is the medical specialty with long history, which deals with the prevention, diagnosis, and treatment of adults patients who have undifferentiated or multi-system disease processes [3]. With the development of internal medicine, independent status for some of the subspecialties seems inevitable. The American Board of Internal Medicine (ABIM) approved requests for subspecialties since 1940s, and many independent subspecialties developed since 1970s [4]. The ABIM is adding new subspecialties, even in the past 2 years [5].

In the current environment of growing demands for higher standards of medical care, new specialties can benefit both patients and physicians. In the U.S. the subspecialties of internal medicine are well integrated in the overall discipline of internal medicine and each one is also unique.The ABIM presently certifies 16 unique subspecialties. In recent years, some subspecialties have been at a third level, with sub-subspecialty developed. The development of a subspeciality and its branching out into sub-subspecialities is influenced by the volume of patients the specialists cater for and how easy it is to further subdivide their care. Cardiology, as the largest subspecialty, has been a good example of the three levels of certification. Certification includes Clinical Cardiac Electrophysiology and Interventional Cardiology [6]. This trend to some extent is reflected from the change of journal numbers in each subspecialty. Although journal numbers in nine subspecialties were increased, cardiac and cardiovascular system disease had the highest increment of 131.2%, hematology had the lowest increment of 45%. This reflected, to some extent, the subspecialties in internal medicine prosper unevenly.

At present, due to lack of unified measurement or evaluation methods, it is difficult to estimate and compare the differences of development trend in various subspecialties among different subspecialties. One measure of a journal’s visibility and accessibility is the impact factor, which is a means of ranking journals by citation analysis [7]. Although there exist some disadvantages and controversial, the impact factor remains an important method for evaluation of journal quality [8].

A journal’s calculated impact factor affects journal prestige, influences authors’ decisions about where to submit their work. Authors consider the impact factor prior to submitting an article to a particular journal and naturally may prefer to submit their papers to journals with large circulation and with high impact factors for publication. Currently, many faculty committees involved in promotions or search of new members rely heavily on impact factors, as do scientific boards of granting institutions. For example, the science ministries in some Asian countries now offer rewards to their scientists if they are able to publish papers in journals with high impact factors [9]. Therefore, an evaluation of average level and the growing change of impact factors in each subspecialty would help to elucidate the academic performance of internal medicine journals.

The results from this study showed a gradual increase in the impact factor of scientific journals in nine subspecialties occurred during the 12-year study period, reflecting the substantial development of all subspecialties in internal medicine during the recent years. A gradual decrease in the journal impact factor less than 2 were found among the subspecialties of infectious disease, cardiac and cardiovascular system disease, gastroenterology and hepatology, hematology, endocrinology and metabolism. This upward trend pattern among these subspecialty journals addressed the improvement of academic publications in these fields. By analyzing the change of journal impact factors from 1998 to 2010, we found allergy had a greatest increase by 121.14% in their median impact factor. The finding from this study suggests that allergy is a young but rapidly growing field.

We evaluated the general condition and development trend of subspecialties by analyzing the average median impact factor, grade level of impact factor as well as the dynamic change. Endocrinology and metabolism journals had the highest level of average median impact factor, the most absolute number of high impact factor(>6) journals, and the least percentage of low impact factor(<2) journals. A gradual decrease in journal impact factor less than 2 were also found in the field of endocrinology and metabolism. All these findings showed that endocrinology and metabolism had the overall top level among internal medicine journals. In contrast, nephrology journals had the lowest level of average median impact factor, the least absolute number of high impact factor(>6) journals, and the highest percentage of low impact factor(<2) journals. We did not found dynamic change in the journal impact factor less than 2 in the field of nephrology. All these clues demonstrated that nephrology stood the overall low level among internal medicine journals.

The reasons for this discrepancy are quite complex. We tried to give some preliminary explanations. Endocrinology and Metabolism is a relative young but rapidly growing field. More and more attention has been paid on this discipline to promote its progression with a relative high level in internal medicine field. The WHO global status report on noncommunicable diseases 2010 showed that noncommunicable diseases are the biggest cause of death worldwide. Diabetes (3%) was among the leader top 5 noncommunicable diseases [10], which are likely to have great clinical importance. More importantly, our results suggested that a causal association existed between journal number or article number and an increase in journal impact factor. Endocrinology and metabolism stood the second position of journal numbers, which is associated with high impact factor.

Nephrology also shows growth, but this is clearly less than other subspecialties. Due to variability in clinical presentation, variability in treatment response, lack of consensus in definitions, difficulty in recruiting patients and high costs of clinical trials, there is a paucity of high quality clinical trials in the field of nephrology, which may be related to the relatively slow development [11]. Another study documented a weak association between journal impact factor and the methodological quality of published studies [12]. According to the analysis of randomized controlled trial (RCT) in internal medicine, a general low level status was found in this field, including endocrinology and metabolism [13]. But nephrology also lags behind compared with all other subspecialties of internal medicine [14]. RCT in nephrology are relatively few. Quantity and quality of trials in nephrology is of major concern and has substantial room for improvement. In addition, fewer diagnostic studies were published in nephrology than other areas of internal medicine. The quality of nephrology diagnostic test accuracy studies has not improved substantially during the past 30 years [15]. The proportion of internal medicine residents applying to the various subspecialties has potential impact on the development of the fields. There is a growing concern over the imbalance in the numbers and composition of internal medicine subspecialty training programs. Nephrology is not as attractive to many American graduates. Nephrology is an extremely intellectually challenging specialty and many residents are not interested with [16].

Impact factor is a measure of the average number of citations to recent articles published in journals within a particular period. Higher impact factor depends on the higher quality of publications which produce more cited references but not ordinary bulky publications. The evolution of academic performance bring about increased volume of publishing in the subspecialty. A large amount of journals will be expected to create a competitive environment. Academic competitions promoted development of higher level of journals which could be reflected, to some extent, by the highest impact factor, mean or median impact factor in the fields. Therefore, change in academic performance could be assessed by dynamic change in impact factor over time. In the recent years, longitudinal bibliometric analyses of impact factor have been conducted extensively in many fields to reflect the trend of development [17], [18].

To our knowledge, this is the first paper to describe the dynamic changes of journal impact factors in different subspecialties of internal medicine as well as the possible discrepancy among each others. Our findings could provide some references to appreciate the development trend of the subspecialties in internal medicine. This would help both clinicians and researchers during their research and clinical practice to know about the general status of different subspecialties in internal medicine.

Several confounding factors may have affected the results of this study. For example, (1) scientific papers originated from the subspecialties could be submitted and published in general medical journals (such as the New England Journal of Medicine, Lancet), general internal medicine journals (such as the Annals of Internal Medicine), or journal of geriatric medicine or pediatrics. Basic research papers may submit to journal of basic science. These made the journals retrieved inaccurately and may not accurately reflect the status in the field. Besides, not all journals relevant for a subspecialty are part of the Science Citation Index Expanded (SCIE). Nevertheless, this does not change the results and conclusion of this study as SCIE hold the leading position in bibliographic analysis field with most representative journals been included. (2) Each subspecialty is an arbitrarily-constructed discipline. Many new and emerging subspecialties are cross-disciplinary. An increasing number of general and affiliated specialty society journals make finding the right place for manuscript submission of an article challenging. For example, nephrology contributes to, many other disciplines including endocrinology, cardiology, and rheumatology. That means a substantial portion of papers may be published in non-renal journals [19]. (3) The journal impact factor measures just one aspect of article quality [20], which reflects partly the development of subspecialties in internal medicine. Other factors such as higher visibility of journals, an increase of scientific output overall or higher interest in 'core journals' as defined by the Science Citation Index might be contributing factors as well. A journal’s impact factor is also affected by additional factors [21], such as the journal’s language and country origin [22], the journal’s visibility and accessibility [23], the publications of evidence based medicine and systematic reviews [24]. The maximum impact factors might be relevant to review journals and interdisciplinary papers.

In general, given the findings of our study, development of the subspecialties of internal medicine is uneven. Based on the average median impact factor, grade classification and dynamic changes of journal impact factors, we found some indicators reflecting the overall level and development trend of subspecialties in internal medicine field. It is important not to rely solely on one standard, new and emerging measures of scientific impact, such as h-index, Y-factor [25], are expected to be developed to provide a further objective evaluation method for medical journals.
